# Investigating size effects in graphene–BN hybrid monolayers: a combined density functional theory-molecular dynamics study†

**DOI:** 10.1039/d1ra00316j

**Published:** 2021-03-31

**Authors:** I. S. Oliveira, J. S. Lima, A. Freitas, C. G. Bezerra, S. Azevedo, L. D. Machado

**Affiliations:** Departamento de Física, CCEN, Universidade Federal da Paraíba Caixa Postal 5008 58051-970 João Pessoa PB Brazil; Departamento de Física, Universidade Federal do Rio Grande do Norte 59072-970 Natal RN Brazil leonardo@fisica.ufrn.br

## Abstract

We combine Density Functional Theory (DFT) and classical Molecular Dynamics (MD) simulations to study graphene–boron nitride (BN) hybrid monolayers spanning a wide range of sizes (from 2 nm to 100 nm). Our simulations show that the elastic properties depend on the fraction of BN contained in the monolayer, with Young's modulus values decreasing as the BN concentration increases. Furthermore, our calculations reveal that the mechanical properties are weakly anisotropic. We also analyze the evolution of the stress distribution during our MD simulations. Curiously, we find that stress does not concentrate on the graphene–BN interface, even though fracture always starts in this region. Hence, we find that fracture is caused by the lower strength of C–N and C–B bonds, rather than by high local stress values. Still, in spite of the fact that the weaker bonds in the interface region become a lower fraction of the total as size increases, we find that the mechanical properties of the hybrid monolayers do not depend on the size of the structure, for constant graphene/BN concentrations. Our results indicate that the mechanical properties of the hybrid monolayers are independent of scale, so long as the graphene sheet and the h-BN nanodomain decrease or increase proportionately.

## Abstract 2

We combine Density Functional Theory and Molecular Dynamics to study graphene–BN monolayers ranging from 2 nm to 100 nm. We find that the mechanical properties are independent of scale when we increase graphene and the BN domain proportionately.

## Graphical abstract

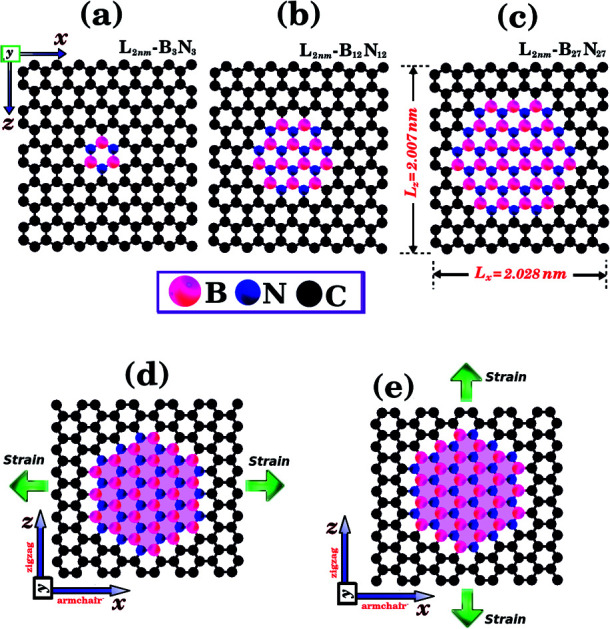

## I. Introduction

Over the past few decades, two-dimensional (2D) materials have been an active research topic due to their exceptional physical and chemical properties, which promise applications in many modern technologies.^1^ Within the family of 2D materials, those composed of sp^2^-hybridized atoms arranged in a honeycomb-like hexagonal lattice stand out, with graphene^2–4^ and hexagonal boron nitride (h-BN)^5–7^ being the two most notable examples. Graphene in its pristine form is a semimetal^8^ with the highest Young's modulus (≈1 TPa) and ultimate tensile strength (≈130 ± 10 GPa) ever measured.^9–12^ On the other hand, h-BN in its pristine form is an insulator (>4 eV) with high resistance to oxidation.^13,14^ The mechanical properties of h-BN are also outstanding, with a Young's modulus of ≈0.665 TPa and an ultimate tensile strength of ≈70.5 ± 5.5 GPa.^9,15–17^ Both graphene and h-BN are materials with a promising future for theoretical/experimental studies and practical applications in nanotechnology.

In spite of their excellent properties, there is evidence indicating that graphene and h-BN are not suited for the construction of certain nanoelectronic devices.^18–21^ The reason for this is that the band gap of graphene (h-BN) is too small (large), leading to devices that do not work properly in an on-off current regime. Given this scenario, continuous efforts have been made in order to predict and synthesize alternative 2D structures. Examples include the B_*x*_C_*y*_N_*z*_ hexagonal hybrid sheets (h-B_*x*_C_*y*_N_*z*_ sheets), which are structures composed of carbon, boron, and nitrogen that have already been synthesized.^22–24^ These materials can be thought of as being a graphene with part of their C atoms replaced by B and N atoms. The properties of h-B_*x*_C_*y*_N_*z*_ sheets are strongly dependent on the arrangement of the C, B, and N atoms, as well as the particular stoichiometry.^25–29^ These sheets exhibit a range of band gaps (<2 eV) that are intermediate between those found in graphene and h-BN.^26–31^ The adjustable band gap of these hybrid 2D materials indicate that they are better candidates than either graphene or h-BN for the production of modern nanoelectronic devices.

An interesting subset of the B_*x*_C_*y*_N_*z*_ sheets are those composed of h-BN nanodomains embedded within a larger graphene sheet (graphene–BN sheets). Structures of this type were synthetized for the first time by Ci *et al.* using the thermal catalytic CVD method.^22^ The reported HRTEM images revealed that most samples had two or three layers, and included large h-BN nanodomains with irregular geometric forms. These novel 2D hybrid materials have physical–chemical properties that can be tailored by controlling the size of the h-BN nanodomains. After this discovery, other experimental methods were developed for the growth of this type of structure.^23,24,32–36^ Concurrently, Manna *et al.* performed DFT calculations to investigate hybrid sheets including h-BN nanodomains of varying size and geometry (hexagonal, rhombus, and triangular).^37^ The authors found that the boundary between the h-BN nanodomain and graphene governs the electronic properties of these structures. Depending on the number of C–B and C–N bonds, the graphene–BN sheets may be metallic, semimetallic, or semiconducting. Similar results were found in other reports.^38–42^

Another common approach to modulate the physical–chemical properties of 2D materials is to use strain engineering, where the properties of a material are modified *via* controlled mechanical deformation.^43–47^ This can be accomplished, for example, through the application of tensile stress.^48–51^ Depending on the direction and magnitude of this stress, the material can be bent, wrinkled, stretched, or even broken. Experimental methods to introduce tensile stress include: (i) depositing the 2D structure on a flexible substrate that can be bent, elongated or shrunk;^52–54^ (ii) depositing the 2D material on top of a hole in a substrate, and then pushing it down with an atomic force microscope (AFM) tip.^55^ All of these findings have contributed to the emergence of a new research area called “straintronics”, where strain engineering methods and strain-induced physical effects are used to develop devices for new technologies.^56,57^ Finally, note that the mechanical properties of a material, such as stiffness and tensile strength, can also be determined through the application of a tensile stress.

Many theoretical studies have investigated the mechanical properties of 2D materials. For example, Zhao *et al.* investigated the mechanical properties of graphene–BN sheets using molecular dynamics (MD) simulations.^58^ These authors considered different sizes, shapes, and amounts of h-BN nanodomains embedded in graphene sheets, and found that hybrid sheets displayed strong plasticity behavior. They also found that the Young's modulus of the hybrid sheets presented values intermediate between those of graphene and h-BN, decreasing as the concentration of h-BN increased. It is hoped that future experimental studies can confirm this prediction. Recently, Azevedo and Kaschny employed DFT calculations to study the mechanical properties of one of the structures considered by Zhao *et al.*, namely, a graphene sheet containing a hexagonal h-BN nanodomain in its center.^59^ In addition to investigating the mechanical properties of the graphene–BN sheets, these authors also studied how the electronic properties of the hybrid sheets varied with increasing strain. Additionally, MD^60^ and DFT studies^61^ investigated the mechanical properties of B_*x*_C_*y*_N_*z*_ hexagonal sheets with atomic arrangements including a large amount of B–C and N–C bonds. The reported values of stiffness and ultimate tensile strength were lower than those reported for structures composed of h-BN nanodomains embedded in graphene sheets.

Still, up to now all simulation studies have only considered small model structures, far smaller than those investigated in experimental studies. And have not considered whether the small unit cell size could affect the obtained results. Note, for instance, that the fraction of B–C and N–C bonds decreases as the structure size increases. In order to verify possible size effects, here we investigated the mechanical properties of square graphene–B_*x*_N_*z*_ sheets with sizes ranging from 2 nm to 100 nm, using molecular dynamics simulations. Furthermore, in order to assess the reliability of the classical potential considered, we performed DFT calculations for the smallest structures considered here and compared the DFT and MD results. Our calculations show that the MD results are reliable, and that the mechanical properties do not depend on the scale of the considered structures, so long as the size of the h-BN nanodomain and the graphene sheet are increased by the same factor.

## II. Computational details and methods

**Fig. 1:**
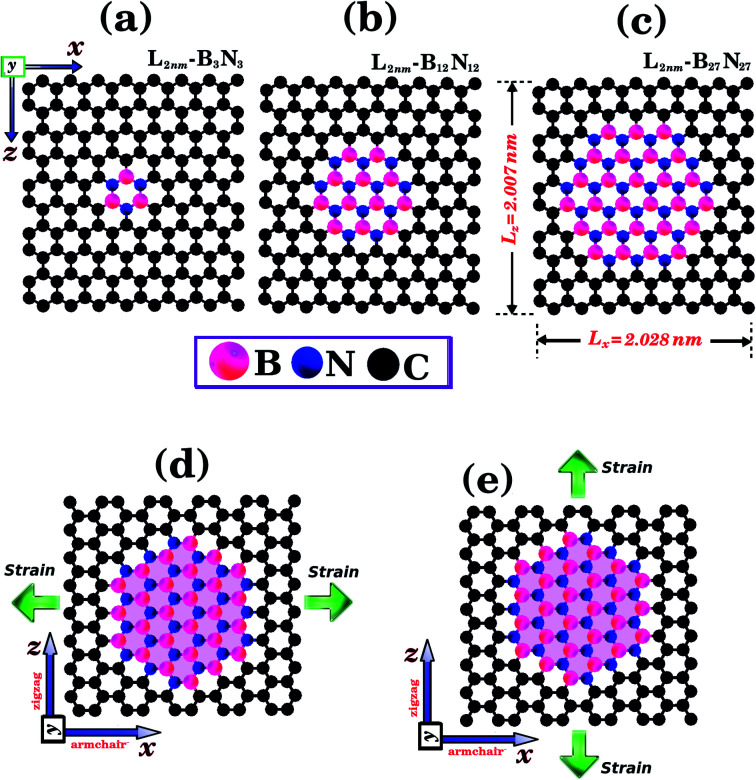
Structures that were investigated using both DFT and MD simulations. They are composed of hexagonal h-BN nanodomains embedded within square graphene monolayers, with side length *L* = 2 nm. The graphene/BN concentrations are: (a) 0.04, (b) 0.15, and (c) 0.34. In (d) and (e) we illustrate the directions where strain is applied to the structure.

**Fig. 2:**
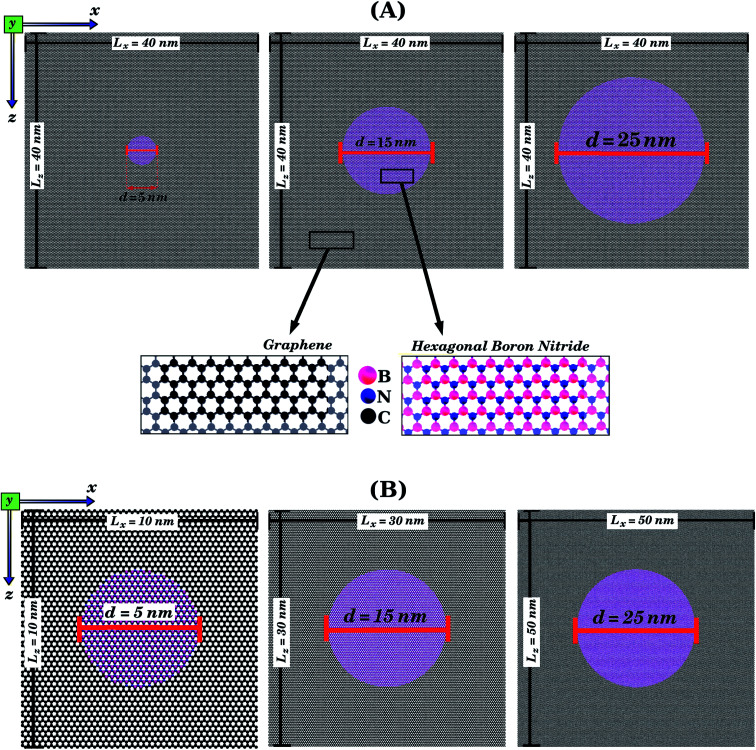
Structures that were investigated using only MD simulations, due to their large size. They are composed of circular h-BN nanodomains (with diameter *d*) embedded within square graphene sheets (with side length *L*). In (A), we illustrate the case where *L* is kept constant while the BN concentration varies. In (B), we illustrate the case where *L* varies while the BN concentration is kept constant. For all structures, we first performed calculations with strain applied to the armchair and then with strain applied to the zigzag direction (see Fig. 1(d) and (e)).

We combined first principles calculations and MD simulations to investigate the mechanical properties of graphene nanosheets containing h-BN nanodomains (graphene–BN sheets). Some of the structures considered here are shown in Fig. 1 and 2. Notice that we considered square graphene sheets in all of our simulations (*L*_*x*_ = *L*_*z*_ = *L*), with side lengths (*L*) ranging from 2 to 100 nm (see Tables 1 and 2). Regarding the geometry of the h-BN nanodomains, we considered hexagonal domains for the smaller structures (displayed in Fig. 1) and circular domains for the larger structures (displayed in Fig. 2). We denote the number of B and N atoms in the nanodomain by *n*_B_ and *n*_N_, and we only considered structures with *n*_B_ = *n*_N_. In the investigated structures, *n*_B_ and *n*_N_ ranged from 3 to 37 588 atoms. Similarly, we denote the number of C atoms in the graphene sheet by *n*_C_, which ranged from 154 to 309 976 atoms. In order to unequivocally identify each structure, it is useful to introduce the following nomenclature. In the present work, each structure is specified by the side length of the sheet *L*_*i*_ (*i* ranges from 2 to 100 nm) and by the molecular formula B_*x*_N_*x*_, which specifies the number of atoms in the domain. For example, *L*_2nm_-B_3_N_3_ stands for the hybrid sheet illustrated in Fig. 1(a), which has 2 nm in length and a h-BN nanodomain with 3 boron and 3 nitrogen atoms. Moreover, for each structure we calculated the atomic fraction of h-BN (*γ*), which is determined by the following equation:

1 *γ* = (*n*_B_ + *n*_N_)/*n*_T_,

where *n*_B_ and *n*_N_ follow the previous definition, and *n*_*T*_ is the total number of atoms. *γ* values for the investigated structures vary from 0.01 to 0.34, as shown in Table 1.

**Table 1:** Calculated mechanical properties of structures illustrated in Fig. 1(a)–(c). *L* is the side length, *γ* is the atomic fraction of BN, *Y* is the Young's modulus, *σ* is the tensile strength, and *ε* is the ultimate strain

	*L* (nm)	*γ*	Armchair	Zigzag	Armchair	Zigzag
			*Y* (GPa)	*Y* (GPa)	*σ* (GPa) [*ε*]	*σ* (GPa) [*ε*]
**DFT calculations**						
*L*_2nm_-B_3_N_3_	2	0.038	843.4	873.8	109.3 [0.22]	96.7 [0.17]
*L*_2nm_-B_12_N_12_	2	0.150	837.3	857.0	105.4 [0.22]	94.1 [0.17]
*L*_2nm_-B_27_N_27_	2	0.338	820.4	810.4	101.1 [0.21]	86.1 [0.14]

**MD simulations**						
*L*_2nm_-B_3_N_3_	2	0.038	956.97	948.50	115.2 [0.19]	107.8 [0.17]
*L*_2nm_-B_12_N_12_	2	0.150	912.59	913.61	101.6 [0.16]	100.7 [0.16]
*L*_2nm_-B_27_N_27_	2	0.338	869.61	856.81	101.5 [0.17]	94.6 [0.15]

**Table 2:** Calculated mechanical properties of structures illustrated in Fig. 2(a) and (b). *L* is the side length, *γ* is the atomic fraction of BN, *Y* is the Young's modulus, *σ* is the tensile strength, and *ε* is the ultimate strain

MD simulations	*L* (nm)	*γ*	Armchair	Zigzag	Armchair	Zigzag
			*Y* (GPa)	*Y* (GPa)	*σ* (GPa) [*ε*]	*σ* (GPa) [*ε*]
*L*_10nm_-B_376_N_376_	10	0.191	908.31	901.14	96.8 [0.14]	100.1 [0.15]
*L*_20nm_-B_1503_N_1503_	20	0.191	907.49	897.72	101.0 [0.14]	100.7 [0.14]
*L*_30nm_-B_3383_N_3383_	30	0.194	906.02	896.84	90.9 [0.12]	88.4 [0.12]
*L*_40nm_-B_376_N_376_	40	0.012	964.86	956.57	97.2 [0.14]	98.4 [0.14]
*L*_40nm_-B_1504_N_1504_	40	0.049	951.71	943.15	96.3 [0.14]	95.5 [0.13]
*L*_40nm_-B_3383_N_3383_	40	0.109	932.84	922.16	91.1 [0.12]	88.1 [0.11]
*L*_40nm_-B_6013_N_6013_	40	0.194	908.38	897.88	96.4 [0.13]	89.6 [0.12]
*L*_40nm_-B_9404_N_9404_	40	0.304	876.81	866.17	88.3 [0.12]	97.6 [0.12]
*L*_50nm_-B_9404_N_9404_	50	0.195	904.89	898.38	87.9 [0.11]	92.4 [0.12]
*L*_100nm_-B_37588_N_37588_	100	0.195	907.38	897.84	93.4 [0.13]	101.4 [0.15]

To determine the mechanical properties of the graphene–BN sheets, we applied a tensile strain along one direction and then calculated the resulting tensile stress to obtain a stress–strain curve. The methodology used to obtain the stress differed in the DFT and MD simulations. In the former, a derivative of the total energy was used in the calculation, while in the latter the forces acting on the atoms were used. Details can be found in the ESI.† In the process used in the DFT calculations, we first increased one side of the simulation box by 1% and then relaxed the other side and the atomic positions until the calculation converged. We then increased the simulation box by 1% again, and repeated the process. For the MD simulations, we increased one direction of the simulation box continuously at a fixed strain rate, and used a barostat to keep the other direction relaxed. Meanwhile, we allowed all atoms to evolve freely. Strain was applied along the armchair (*x*) and zigzag (*z*) directions, as illustrated in Fig. 1(d) and (e). Additional details are discussed below.

The first principles calculations are based on density functional theory (DFT),^62^ as implemented in the SIESTA code.^63,64^ The exchange-correlation energy is expressed within the generalized gradient approximation (GGA),^65^ in the form of the Perdew–Burke–Ernzerhof (PBE) functional. We used the double-*ζ* polarized basis set (DZP), with core electrons described by norm-conserving Troullier–Martins pseudopotentials,^66^ in the Kleinman–Bylander factorized form.^67^ The optimization of atomic positions was allowed to proceed until the force on each atom was less than 0.1 eV Å^−1^. A convergence criterion was adopted where self-consistency is achieved when the maximum difference between the output and the input of each element of the density matrix, in a self-consistent field cycle, is less than 10^−4^ eV. Charge density was represented in the real space by a cutoff of 150 Ry for the grid integration. The Brillouin zone was sampled using a 10 × 1 × 10 *k*-point mesh within the Monkhorst and Pack scheme. We adopted a rectangular unit cell. When the tensile strain is applied to one direction, periodic boundary conditions are imposed in the other directions. A vacuum region of 100 Å was added along the *z* direction to avoid artificial interaction between neighboring images.

The MD simulations were carried out using the Large-Scale Atomic/Molecular Massively Parallel Simulator (LAMMPS) code.^68^ The interactions between B, C, and N atoms were described with the Tersoff potential,^69^ using the parameters adjusted by Kinaci *et al.*^70^ Our MD simulations were performed using a reasonably small timestep of 0.1 fs, and proceeded in three steps:

(1) We first evolved the system for 2 × 10^5^ steps in the NPT ensemble. Nose–Hoover thermostats and barostats^71–73^ were used to set temperature and pressure values to 10 K and 0 Pa.

(2) The thermostat was turned off and then the system was evolved for 2 × 10^5^ steps in the NPH ensemble, using the same barostat described above to set the pressure to 0 Pa. Note that the barostat was only applied to the planar direction that is not strained in the following simulation step.

(3) In the final step we maintained the barostat configuration described in Step 2, and then pulled the system for 3 × 10^6^ steps using a strain rate of 10^−6^ fs^−1^ (for a total strain of 30%).

Initially, we performed DFT calculations and MD simulations to determine the mechanical properties of the small hybrid sheets (*L* = 2 nm) shown in Fig. 1(a)–(c). Then, we performed only MD simulations for the larger hybrid sheets (10 ≤ *L* ≤ 50 nm) shown in Fig. 2(a) and (b). In Fig. 2(a), we illustrate the case in which *L* is kept constant and the graphene/BN concentration is variable. In (b), we illustrate the case in which *L* is variable and the graphene/BN concentration is kept constant. For the latter, we also performed MD simulations using a larger structure with *L* = 100 nm. For all structures, we obtained stress–strain curves by applying a tensile strain along the zigzag or the armchair direction. Next, for the various stress–strain curves we calculated the slope of the linear region to determine the Young's modulus (*Y*) of each structure. The tensile strength (*σ*) and the ultimate strain (*ε*) are taken at the point where the stress reaches its maximum value. The calculated values of *Y*, *σ*, and *ε* are presented in Tables 1 and 2. Additionally, we analyzed how the graphene/BN concentration and the structure size influenced the obtained results.

## III. Results and discussion

**Fig. 3:**
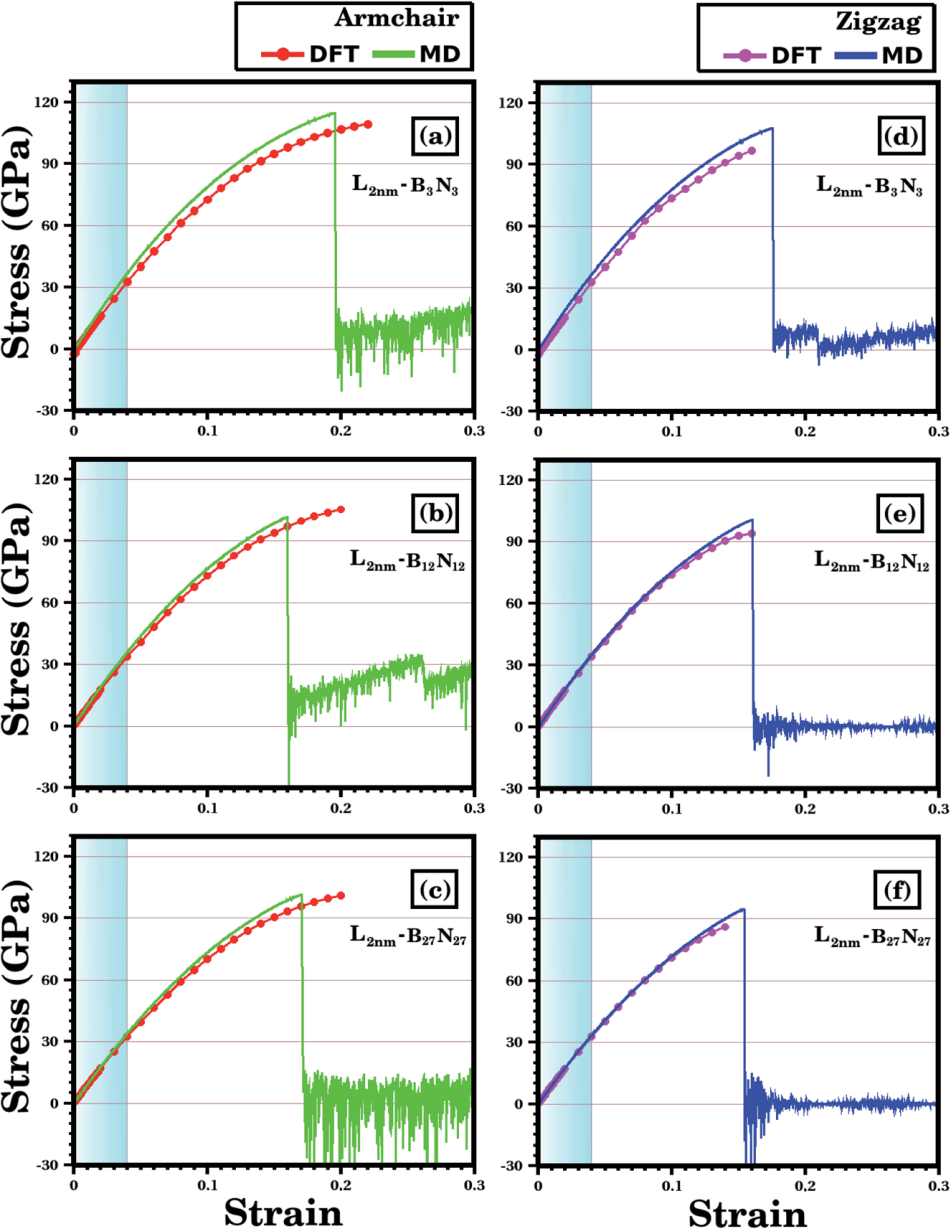
Stress–strain curves obtained through DFT and MD simulations, for the structures shown in Fig. 1. In (a)–(c) strain is applied to the armchair direction; while in (d)–(f) strain is applied to the zigzag direction. In the DFT results, the solid circles indicate the data points, whereas the line is a guide to the eye. In the MD results, the number of data points is large, and a line is used to connect adjacent points. The light blue region corresponds to the elastic region.

We analyze first the mechanical properties of *L*_2nm_-*B*_3_*N*_3_, *L*_2nm_-*B*_12_*N*_12_, and *L*_2nm_-*B*_27_*N*_27_, illustrated in Fig. 1(a)–(c). The obtained stress–strain curves for both the armchair and zigzag directions are shown in Fig. 3. In all cases, note the near linear stress response for strain values between 0 and 0.04 (or 4%). This corresponds to the elastic deformation region, which is highlighted in light blue in Fig. 3(a)–(f). Notice the good agreement between the DFT and MD results in this region. On the other hand, as strain values increase above 4%, the stress response becomes increasingly non-linear. This continues until fracture occurs, at strain values ranging between 14% and 22%. Analyzing the results, we notice that the tensile strength is slightly larger in the armchair direction than in the zigzag direction. In addition, note that for higher strain values the agreement between the DFT and MD results is not as good; however, the difference is still small, particularly in the zigzag direction. In the armchair direction, the main discrepancy observed is that fracture begins in the MD simulations at noticeably smaller strain values. We attribute the observed differences to the distinct methodologies employed in the DFT and MD simulations. For example, in the latter we used non-zero temperatures. Finally, we find that the stress–strain curves obtained here for the hybrid sheets are similar to those reported for other 2D materials.^43,44^

The mechanical properties of *L*_2nm_-*B*_3_*N*_3_, *L*_2nm_-*B*_12_*N*_12_, and *L*_2nm_-*B*_27_*N*_27_ are summarized in Table 1. Comparing the results obtained for the zigzag and armchair directions, we find that the hybrid sheets are weakly anisotropic, with Young's modulus and tensile strength values varying by ∼2.4% and ∼12.5%, respectively. This anisotropy in the mechanical properties was reported for both h-BN^74^ and graphene.^75^ In the case of graphene, this behavior was attributed to the hexagonal structure of its unit cell.^75^ Our results also show that Young's modulus (*Y*) and tensile strength (*σ*) values decrease with increasing concentrations of h-BN, regardless of the direction of applied strain. These results are in good agreement with those reported by Zhao *et al.*^58^ and Azevedo and Kaschny.^59^ Moreover, our DFT and MD results show that the hybrid sheets present *Y* and *σ* values intermediate between those of graphene and h-BN.^9,10,15^ Finally, our simulation results reveal that the tensile strength of the hybrid sheets is about 22.2% lower than that of graphene (130 GPa) and 23.6% higher than that h-BN (70.5 GPa).

According to the DFT calculations, the tensile stress applied to *L*_2nm_-*B*_3_*N*_3_, *L*_2nm_-*B*_12_*N*_12_, and *L*_2nm_-*B*_27_*N*_27_ reaches a maximum for strain values around 21% for the armchair and 15% for the zigzag direction. For higher strain values, the material undergoes fracture (see Fig. 5), and from this point on the DFT calculations no longer converge. Regarding the obtained fracture patterns, we find that they depend mostly on the applied strain direction. For a strain applied along the zigzag direction, we observe that the material breaks in half for any graphene/BN concentration (see Fig. 5(c) and (d)). On the other hand, for a strain applied along the armchair direction, we observe multiple diagonal fractures extending from the edges to the center of the material (see Fig. 5(a)). The same fracture pattern was observed for *L*_2nm_-*B*_3_*N*_3_. Note that for the *L*_2nm_-*B*_27_*N*_27_ structure we were not able to observe the complete fracture of the material, due to convergence issues (see Fig. 5(b)).

**Fig. 4:**
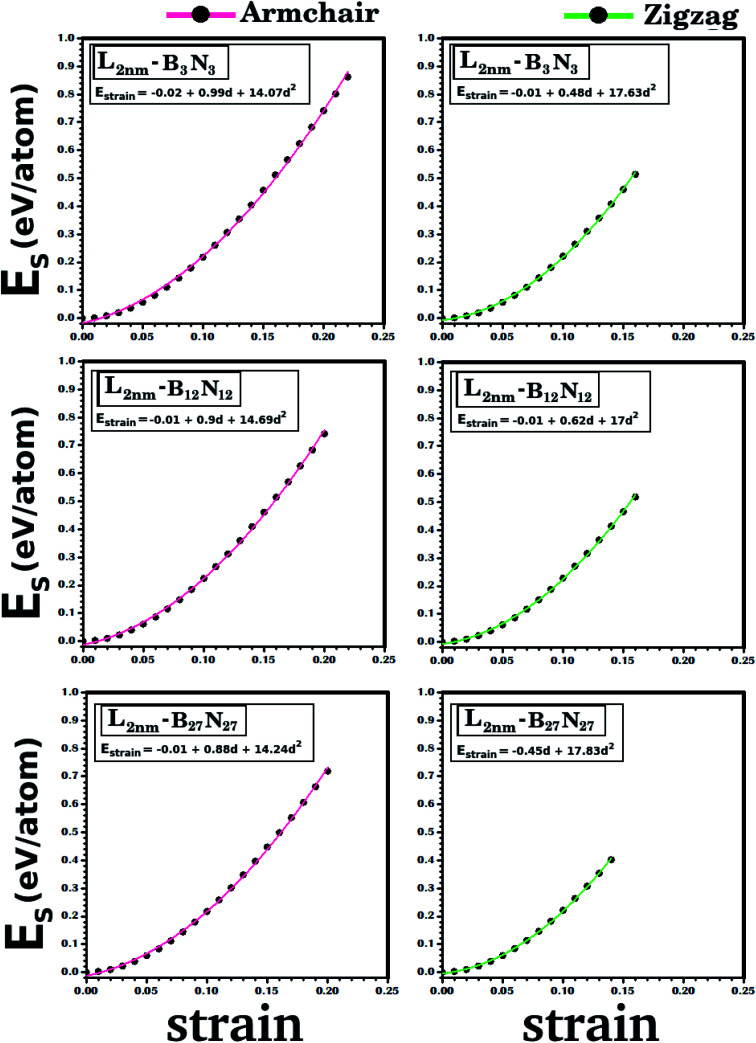
Strain energy (*E*_strain_) plotted against the strain, for the structures illustrated in Fig. 1. Results for the armchair (zigzag) direction are presented in the left (right) column. The solid line indicates the obtained quadratic fit. The coefficients for each fit are presented above its corresponding curve.

**Fig. 5:**
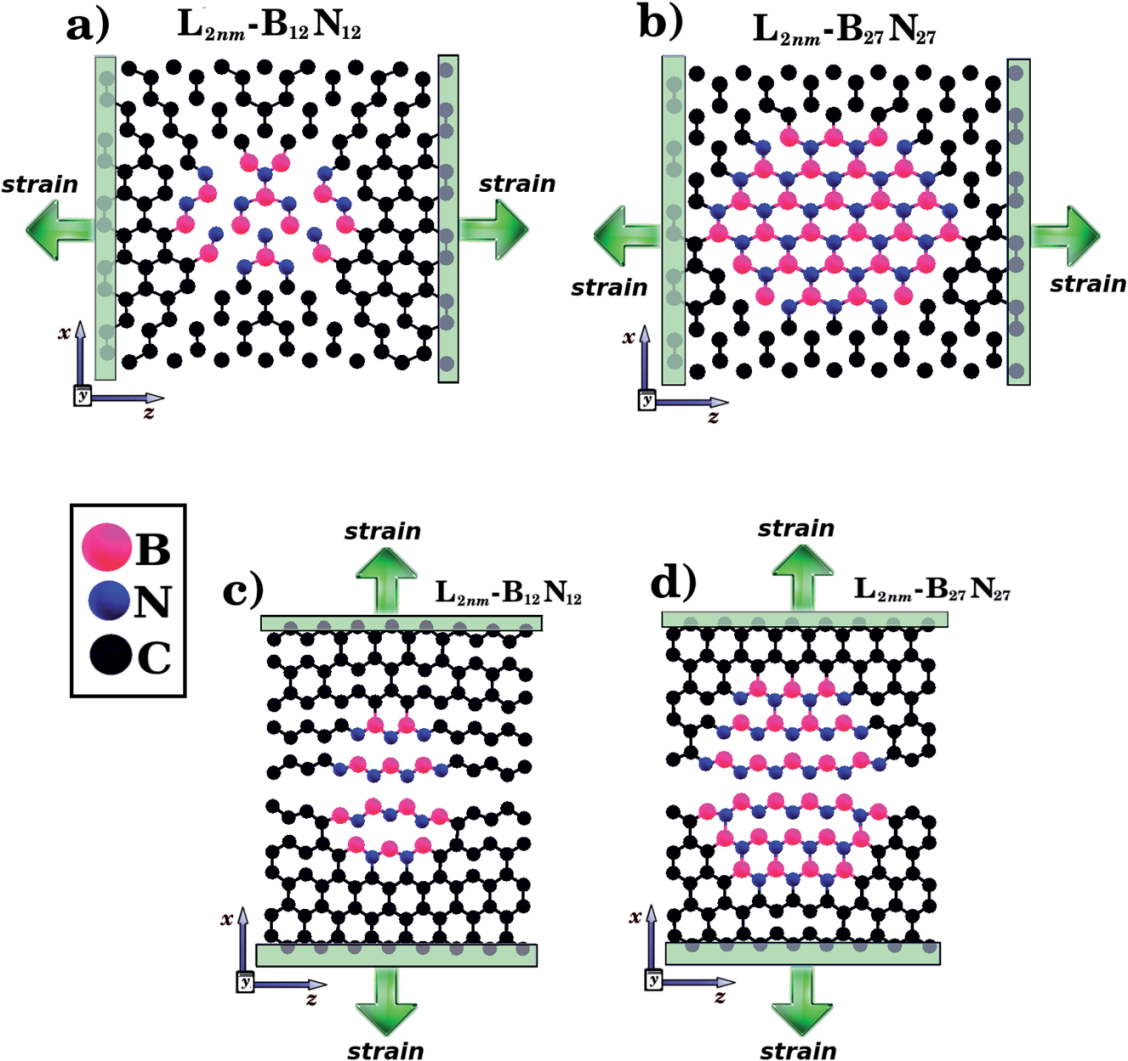
Snapshots from the DFT calculations, detailing the fracture patterns obtained for *L*_2nm_-B_12_N_12_ and *L*_2nm_-B_27_N_27_. Strain was applied to the armchair direction in (a) and (b) and to the zigzag direction in (c) and (d).

To finish the discussion of the DFT results, note that it is possible to obtain the strain energy (*E*_strain_) using the following equation:

2 *E*_strain_ = *E* − *E*_0_,

where *E* and *E*_0_ are the total energies calculated for stretched and unstretched hybrid sheets, respectively. In Fig. 4, we display the variation of the strain energy with strain. The fitting curves exhibit the parabolic behavior that is commonly found in strained 2D materials.^9,59^

**Fig. 6:**
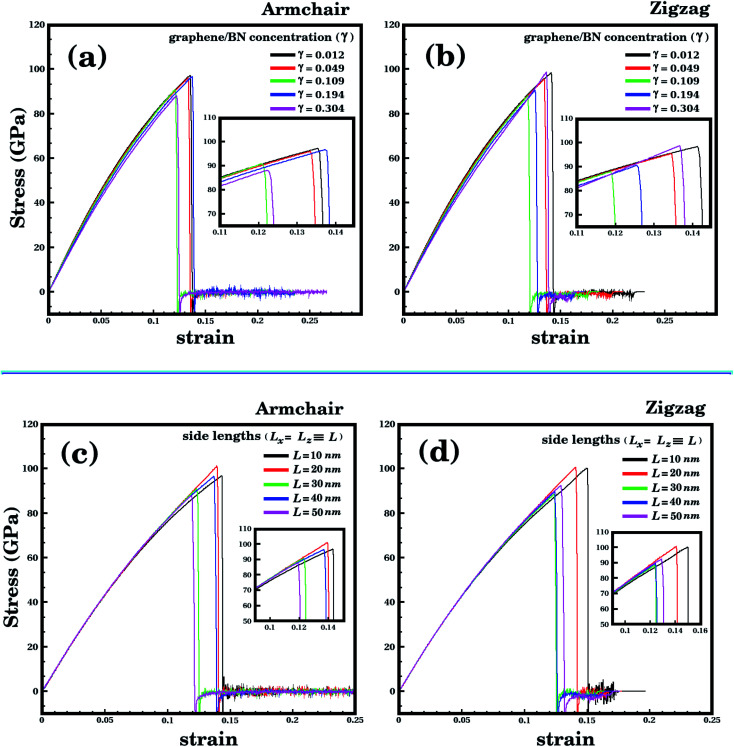
Stress–strain curves obtained using MD simulations. (a) and (b) show results for the case where the BN concentration varies while the side length is kept constant (*L* = 40 nm). (c) and (d) show results for the case where the side length varies while the BN concentration is kept constant (*γ* = 0.19).

Let us now discuss the mechanical properties of the larger hybrid sheets (10 ≤ *L* ≤ 50 nm), which are shown in Fig. 2. Due to their large size, only MD simulations were used to investigate their mechanical properties. Recall that our previous comparison of MD and DFT results for smaller structures indicate that the MD results are reliable, particularly in the elastic deformation region. We consider two types of structures: (i) sheets with constant size (*L* = 40 nm) but varying domain size (*γ* ranging from 0.01 to 0.30, see Table 2); (ii) sheets with constant graphene/BN concentration (*γ* ≈ 0.19), but varying size (10 ≤ *L* ≤ 50 nm). The stress–strain curves obtained for case (i) are shown in Fig. 6(a) and (b) and for case (ii) are shown in Fig. 6(c) and (d). Our results indicate that the linear region for the larger structures extends from 0 to 3%. Unlike the smaller structures, where the tensile strength was always higher in the armchair direction, for some of the larger structures the tensile strength is higher in the zigzag direction (structure *L*_10nm_-*B*_376_*N*_376_ is an example). However, for other structures, such as *L*_20nm_-*B*_1503_*N*_1503_, the tensile strength is still higher in the armchair direction. Finally, note that the stress suddenly drops after reaching its maximum value, due to the fracture of the hybrid sheet.

The mechanical properties of the larger hybrid sheets are summarized in Table 2. We find that the Young's modulus (*Y*) is sensitive to the graphene/BN concentration, but not to the size of the structure (for constant *γ*). Also, notice that *Y* values are higher in the armchair than in the zigzag direction, for all sheet sizes considered. Still, the difference is small, ranging from 0.78% to 11.86%. And, as expected, the tensile strength of the hybrid sheets is considerably lower than that of graphene.

**Fig. 7:**
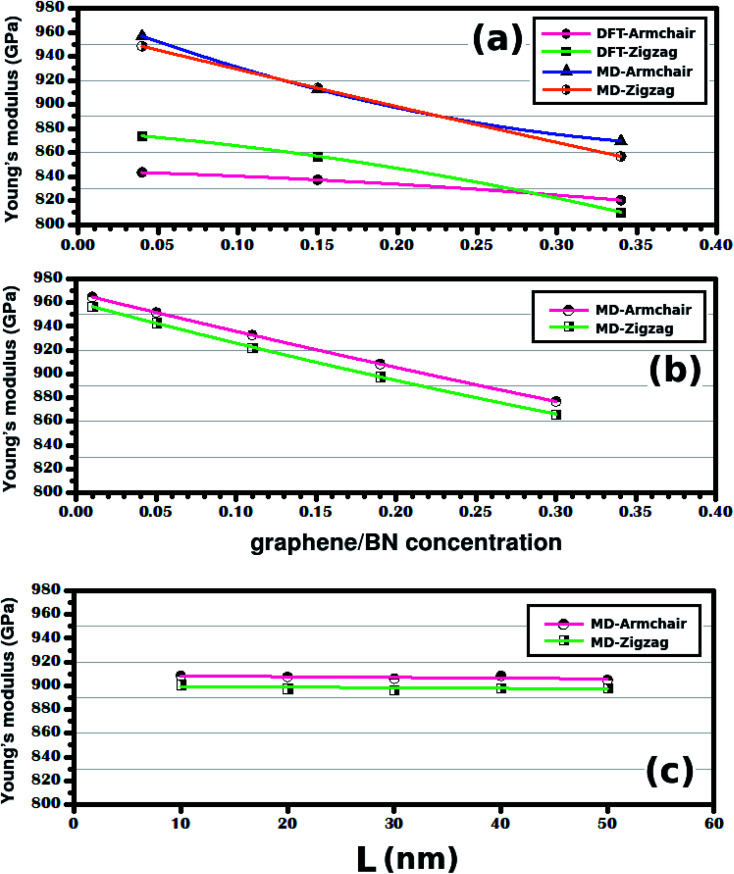
In (a) we have the Young's modulus of the structures shown in Fig. 1, plotted against the graphene/BN concentration. In (b) we have the Young's modulus of the structures shown in Fig. 2(a), plotted against the graphene/BN concentration. In (c) we have the Young's modulus of the structures shown in Fig. 2(b), plotted against the side length *L*.

Let us now analyze results for the case where the concentration is changed while the sheet size is kept constant. Fig. 7(b) shows the Young's modulus variation as a function of the atomic fraction of BN (*γ*). Note that *Y* decreases as *γ* increases for the range of investigated values. The same general behavior was also observed for the smaller hybrid sheets, as shown in Fig. 7(a). In particular, observe that the trend is the same in the DFT and MD results, although *Y* values differ by ∼10%. Finally, note that we also performed calculations using different temperatures and strain rates, to verify the validity of these results. Overall, we observed that the trend remained the same for all temperatures and strain rates, although we also found that the Young's modulus decreased as the temperature and the strain rate increased. These results are detailed in the ESI.†

On the other hand, when the graphene/BN concentration remains constant, the Young's modulus also stays constant (see Fig. 7(c)). We find that *Y* is equal to ∼907 GPa in the armchair and ∼898 GPa in the zigzag direction for *γ* ≈ 0.19. To confirm that the Young's modulus does not depend on the size of the hybrid sheet, we also considered a very large hybrid sheet, with *L* = 100 nm. Results for this structure confirm that the Young's modulus does not depend on the size of the considered sheet for constant *γ* (see Table 2). We also investigated whether our conclusions depend on the shape of the h-BN nanodomain, by performing calculations with large hybrid sheets with hexagonal domains. Overall, we found that our conclusions remained valid in this instance. Results for these simulations can be found in the ESI.† Finally, the simulation results showed that the tensile strength of the hybrid sheets is about 27.3% lower than that of graphene (130 GPa) and 34.1% higher than that of h-BN (70.5 GPa). Still regarding the tensile strength, we found no clear dependence of this quantity with either sheet size or atomic fraction of h-BN. These results are presented in Table 2 and are discussed in more detail in Fig. S5 of the ESI.†

**Fig. 8:**
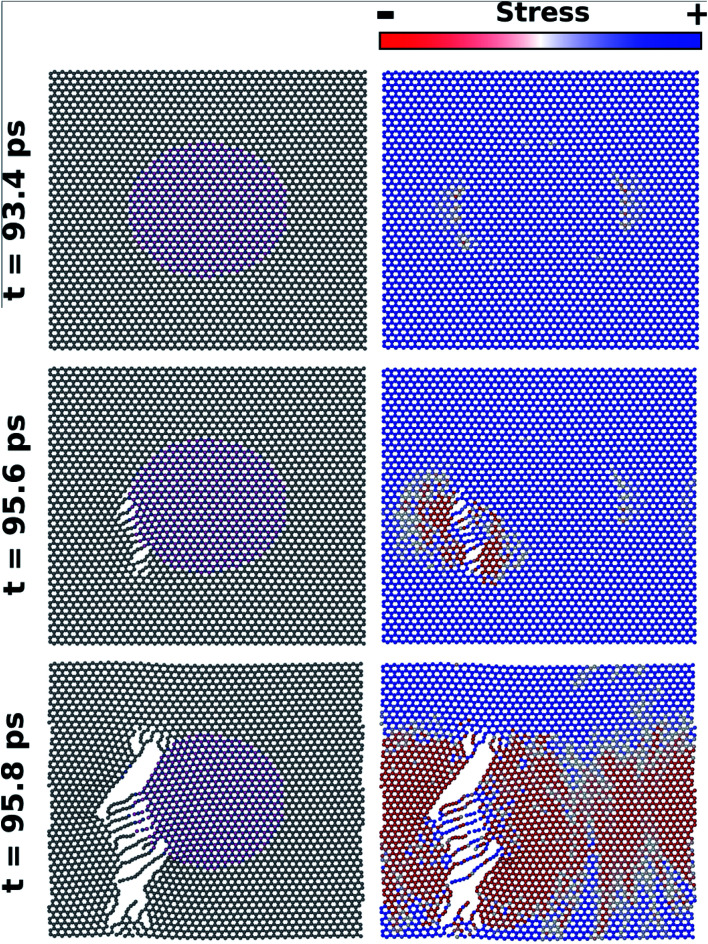
Snapshots from a MD simulation where strain was applied to *L*_10nm_-B_376_N_376_ along the armchair direction. On the left, we have the atomic configuration for different times; on the right, we have the matching stress distribution. For the latter set of images, red corresponds to low, white to intermediate, and blue to high stress. A crack forms in the interface between graphene and the h-BN nanodomain, and quickly propagates.

Fig. 8 shows MD results for the time evolution of the fracture process in the *L*_10nm_-B_376_N_376_ hybrid sheet, under tensile loading in the armchair direction. In this figure, on the left we display the atomic configuration of the system and on the right we display the corresponding stress distribution for a given time. Note that time is equal to zero when strain is first introduced in the system. In the images where stress was used to color the atoms, red corresponds to low, white to intermediate, and blue to high stress values. The stress distribution prior to the fracture of the sheet can be observed in the figures obtained at *t* = 94 ps. At this time, observe that stress is slightly lower at carbon atoms near the h-BN nanodomain. To understand this result, recall that the Young's modulus of graphene is higher than that of h-BN.^58^ Hence, B–N bonds in the domain region tend to stretch more than the adjacent C–C bonds along the armchair direction, so that stress is lower in these C–C bonds. For graphene regions above and below the nanodomain, all bonds along the armchair direction are C–C bonds, so that stress is distributed uniformly.

Still, even though stress is slightly lower near the nanodomain, the fracture actually starts at the interface between graphene and h-BN – see the images obtained at *t* = 95 ps. This result is easy to understand: although stress is not higher at the interface, the B–C and C–N bonds in this region are weaker than the B–N and C–C bonds elsewhere. Hence, in all MD simulations fracture always starts at the interface. After the first bonds rupture, the fracture grows very quickly in the vertical direction, eventually tearing the hybrid sheet (see Fig. 8). As the fracture grows, observe that tension in the sheet is released, and that stress gradually decreases. Also, note that during the fracture process atomic chains are formed. This result is in agreement with those obtained by Zhao *et al.*^58^ and with the experimental process of carbon atomic chain formation from graphene.^76^ In addition, it is important to remark that in all MD simulations the fracture process of the hybrid sheets occurred in a similar way to that described above for *L*_10nm_-*B*_376_*N*_376_. Finally, note that the fracture process is different in the DFT and MD simulations. Regarding the latter, note that the structures are being evolved in time, so that the expected behavior is the fracture growth from an initial crack. On the other hand, there is no time evolution in the DFT calculations, precluding fracture growth. Final structures with lower energy are favored in this instance.

## IV. Conclusions

In summary, we combined DFT and MD simulations to investigate the mechanical properties of graphene sheets containing h-BN nanodomains (graphene–BN sheets). We considered hybrid sheets of varying atomic composition and size (ranging from 2 nm to 100 nm). The stress–strain curves obtained for the smaller structures (*L* = 2 nm) indicate good agreement between the DFT and MD results. For all the considered structures and methods, we found that the Young's modulus decreased as the h-BN concentration increased. In addition, simulation results showed that the tensile strength of the hybrid sheets are ∼15–20% lower than that of graphene. Furthermore, we found that the ultimate strain of the graphene–BN sheets was lower than that of graphene or h-BN, but still greater than 10%. This result indicates that the graphene–BN sheets are well-suited for strain engineering, as they can withstand large strain values. We also found that the mechanical properties of the hybrid sheets are weakly anisotropic. By analyzing the spatial distribution of stress in the hybrid sheets before fracture, we found a rather uniform stress distribution, with slightly lower values in carbon atoms near the h-BN nanodomain. In spite of that, fracture always started at the graphene–BN interface, as the weaker B–C and N–C bonds required lower stress values to break. Finally, we found that the Young's modulus does not depend on the scale of the considered structure, remaining the same so long as the graphene/BN concentration remained constant. This finding indicates that the results described here should remain valid for even larger graphene–BN sheets, like those synthesized in experiments.

## Conflicts of interest

There are no conflicts to declare.

## Supplementary Material

RA-011-D1RA00316J-s001
