# Supplementary material for: Investigating size effects in graphene–BN hybrid monolayers: a combined density functional theory-molecular dynamics study
Source: RSC Adv. 2021 Mar 31;11(21):12595–606. doi: 10.1039/d1ra00316j (PMC8697127; doi:10.1039/d1ra00316j)
Supplement: RA-011-D1RA00316J-s001 [file RA-011-D1RA00316J-s001.pdf]

Electronic Supplementary Material (ESI) for RSC Advances.
This journal is © The Royal Society of Chemistry 2021

# Electronic Supplementary Information: Investigating size effects in graphene-BN hybrid monolayers: a combined Density Functional Theory-Molecular Dynamics study

I. S. Oliveira,[1] J. S. Lima,[2] S. Azevedo,[1] A. Freitas,[2] C. G. Bezerra,[2] and L. D. Machado[2,*]

[1] *Departamento de Física, CCEN, Universidade Federal da Paraíba, Caixa Postal 5008, 58051-970, João Pessoa, PB, Brazil.*

[2] *Departamento de Física, Universidade Federal do Rio Grande do Norte, 59072-970, Natal, RN, Brazil.*

(Dated: March 5, 2021)

**CONTENTS**

Stress definitions 3

Dependence of the Young's modulus with Temperature 4

Dependence of the Young's modulus with the loading rate 5

Dependence of the Young's modulus with the nanodomain shape 6

Dependence of the tensile strength with the side length L and the graphene/BN concentration 8

## STRESS DEFINITIONS

The Stress definition used in the Density Functional Theory (DFT) part of this article follows the description given in other papers in this field [1, 2]. Namely, the stress is obtained using

$$\sigma = \frac{1}{V_o}\frac{\partial U_s}{\partial \epsilon}, \tag{1}$$

where $U_o$ is the initial volume of the structure and $U_s$ is the strain energy, which is related to the total energy as $U_s = U_T(\epsilon) - U_T(\epsilon = 0)$. In this case, $U_T(\epsilon)$ is the total energy for strain $\epsilon$, while $U_T(\epsilon = 0)$ is the total energy for the system without strain. We can also calculate the Young's modulus from the energy $U_s$,

$$Y = \frac{1}{V_o}\frac{\partial^2 U_s}{\partial^2 \epsilon}\ . \tag{2}$$

In the Molecular Dynamics (MD) part of this manuscript, we used the stress definitions given in reference [3]. In this approach, the global stress tensor $\mathbf{P}$ for a collection of $N$ particles in a volume $V$ is given by

$$\mathbf{P}V = \langle \sum_{i=1}^{N} m_i \mathbf{v}_i \otimes \mathbf{v}_i + \mathbf{W}(\mathbf{r}^N) \rangle. \tag{3}$$

In the above expression, the angle brackets indicate an ensemble average; $m_i$ and $v_i$ correspond to the mass and velocity of the $i$th particle, respectively; and the symbol $\otimes$ indicates the direct tensor product. $\mathbf{W}(\mathbf{r}^N)$ is the global virial tensor, which is given by

$$\mathbf{W}(\mathbf{r}^N) = \sum_{\mathbf{n} \in Z^3} \sum_{i=1}^{N} \mathbf{r}_{i\mathbf{n}} \otimes \mathbf{F}'_{i\mathbf{n}}, \tag{4}$$

where $\mathbf{r}$ and $\mathbf{F}$ indicate for a given atom its position and the force acting on it. Special care is taken in this formulation to ensure that it is applicable for systems described by arbitrary many-body potentials under periodic boundary conditions. Further details can be found in the aforementioned article [3].

## DEPENDENCE OF THE YOUNG'S MODULUS WITH TEMPERATURE

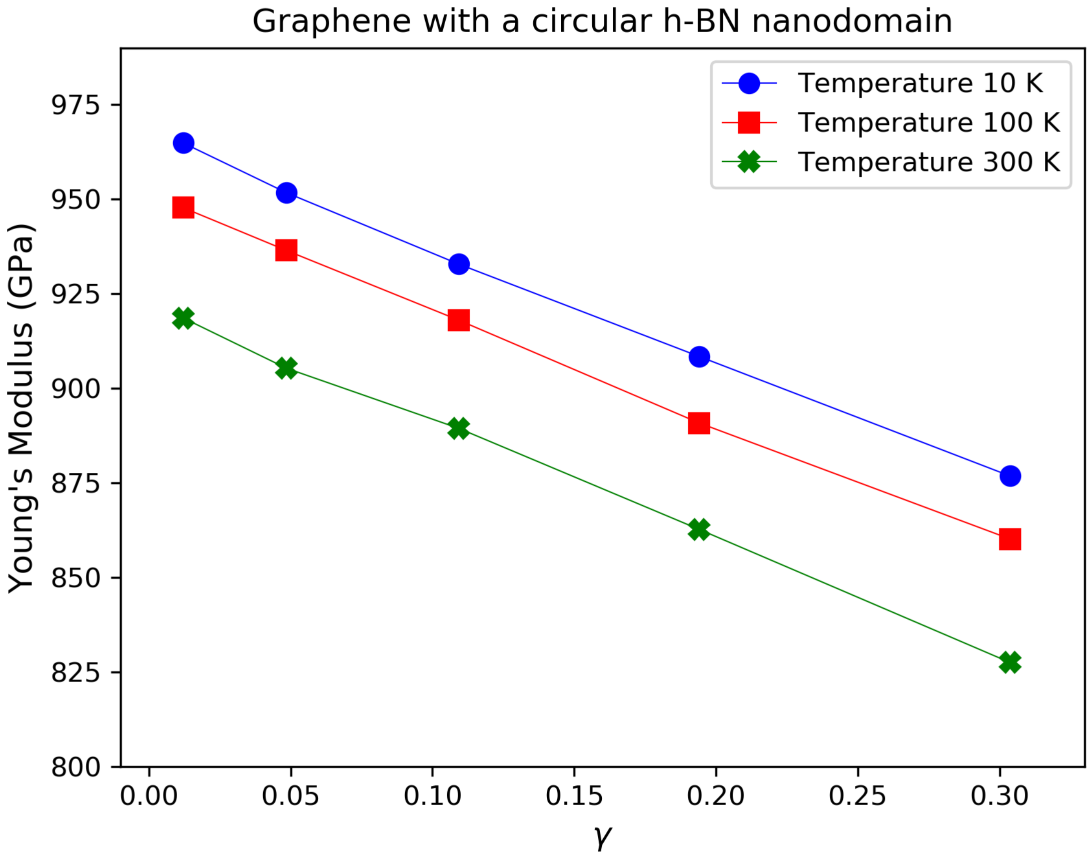


FIG. S1. Young's modulus ($Y$) for graphene with a circular h-BN nanodomain, for different temperatures and graphene/BN concentrations ($\gamma$). Observe that the Young's modulus decreases as the temperature increases. However, the trend regarding the variation of $Y$ with $\gamma$ remains the same for all temperatures. A loading rate of $1 \times 10^{-6}$/fs was used in these simulations.

## DEPENDENCE OF THE YOUNG'S MODULUS WITH THE LOADING RATE

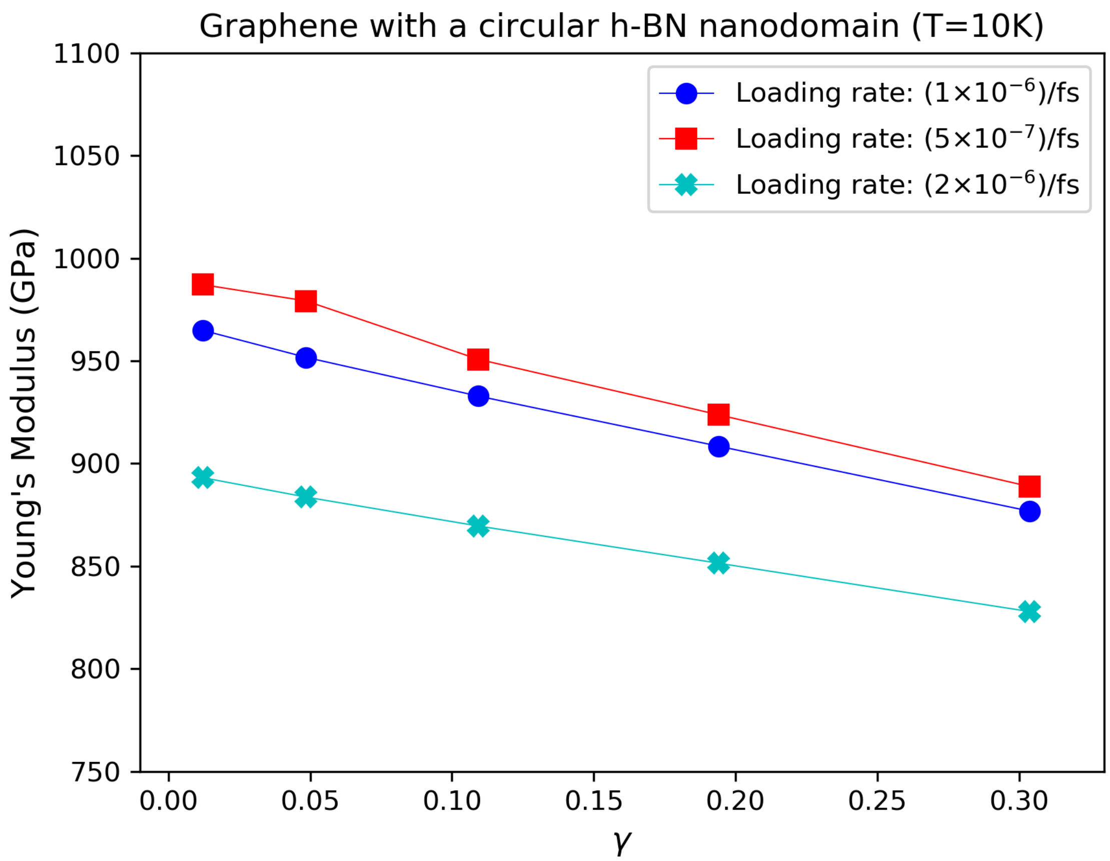


FIG. S2. Young's modulus ($Y$) for graphene with a circular h-BN nanodomain, for different loading rates and graphene/BN concentrations ($\gamma$). Observe that the Young's modulus decreases as the loading rate increases. However, the trend regarding the variation of $Y$ with $\gamma$ remains the same for all the loading rates. A temperature of 10 K was used in these simulations.

## DEPENDENCE OF THE YOUNG'S MODULUS WITH THE NANODOMAIN SHAPE

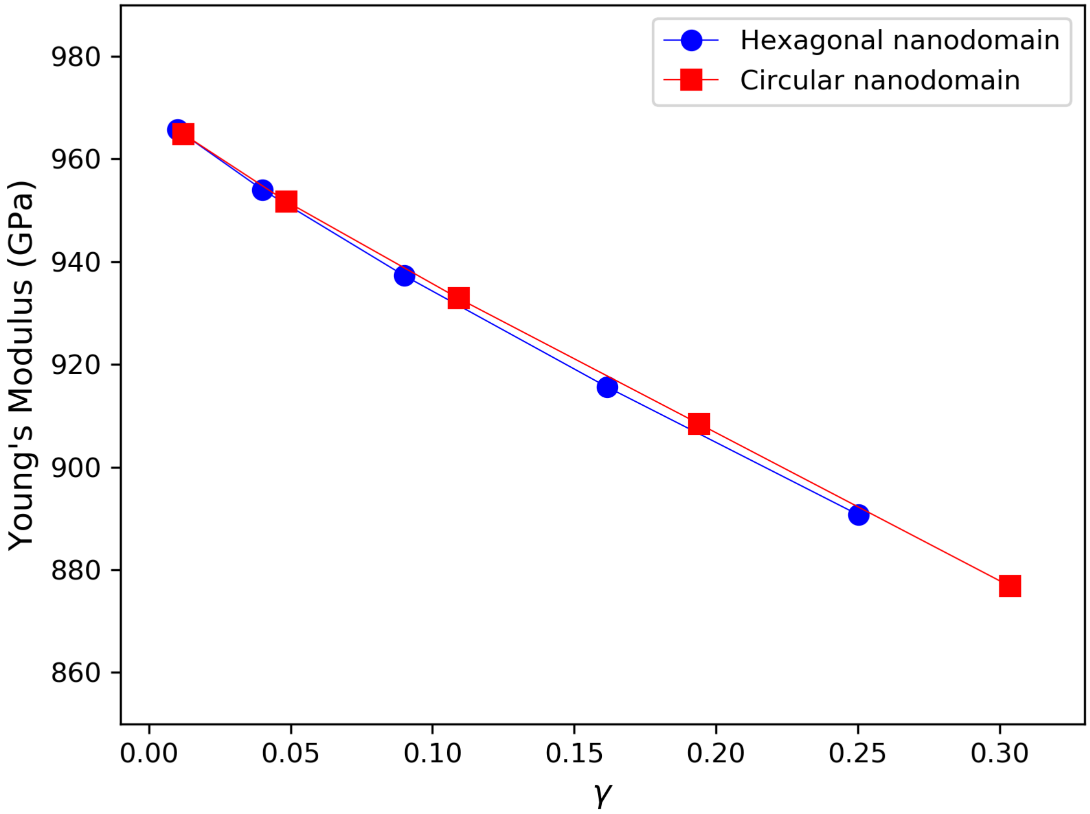


FIG. S3. Young's modulus ($Y$) for graphene with a circular or hexagonal h-BN nanodomain, for different graphene/BN concentrations ($\gamma$). Observe that all results fall roughly within the same line, regardless of the nanodomain shape. A loading rate of $1 \times 10^{-6}$/fs and a temperature of 10 K were used in these simulations.

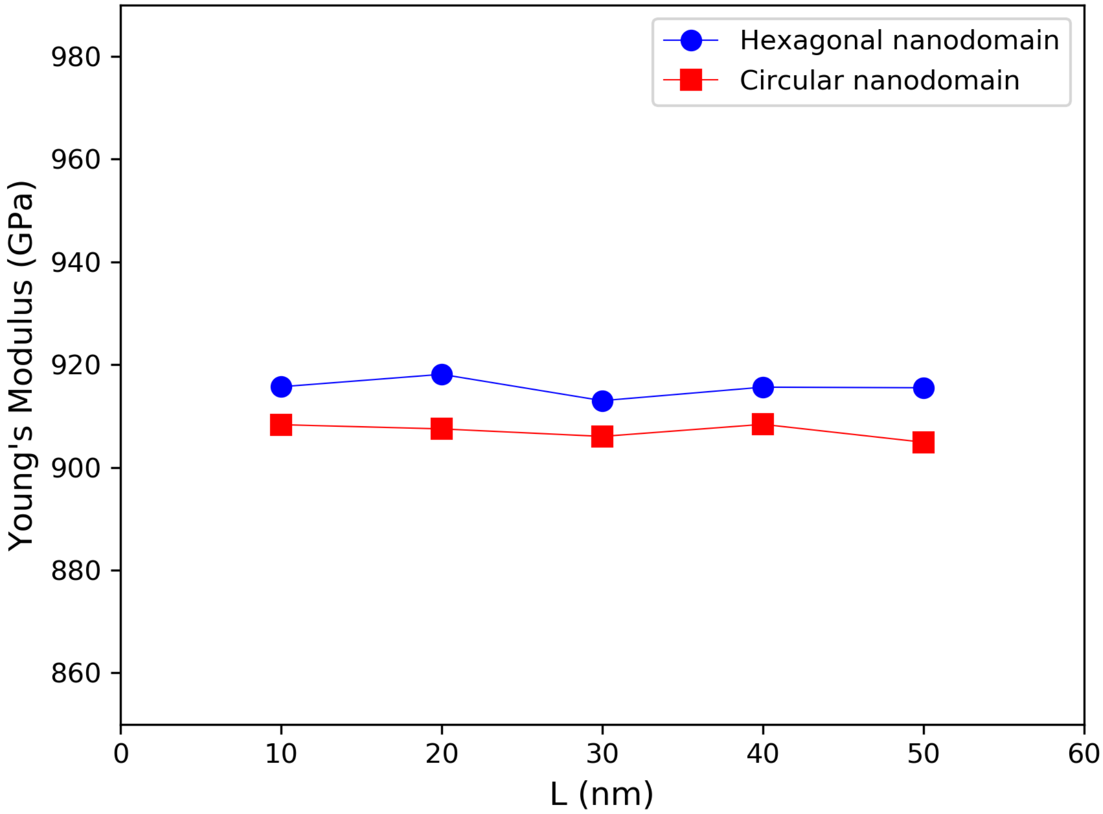


FIG. S4. Young's modulus for graphene with a circular or hexagonal h-BN nanodomain, for different graphene sheet sizes. For all cases, the circle diameter is half the graphene sheet length, and the hexagon length is equal to the circle radius. Structures with circular (hexagonal) nanodomains have a h-BN concentration of $\gamma \approx 0.19$ ($\gamma \approx 0.16$). Hence, hexagonal nanodomains present slightly higher Young's modulus values. A loading rate of $1 \times 10^{-6}$/fs and a temperature of 10 K were used in these simulations.

## DEPENDENCE OF THE TENSILE STRENGTH WITH THE SIDE LENGTH L AND THE GRAPHENE/BN CONCENTRATION

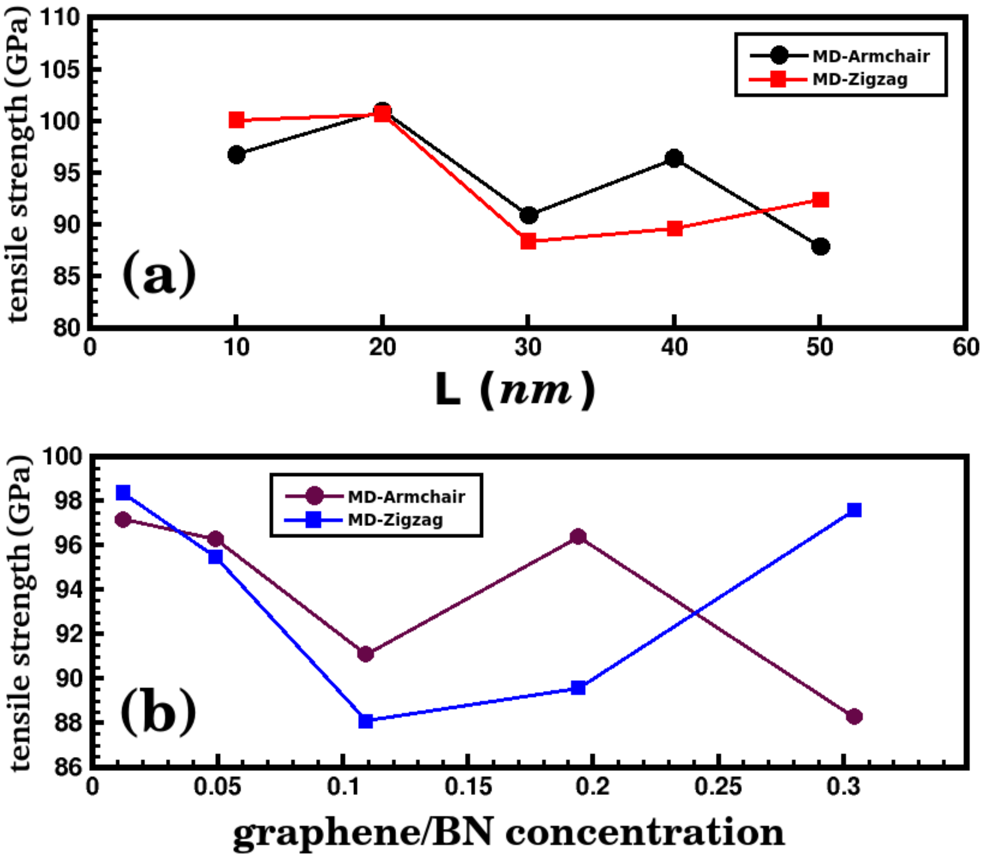


FIG. S5. In (a) we have the tensile strength for the structures shown in Fig. 2(a), plotted against the side length L. In this case, we find that in the armchair direction the tensile strength appears to present an oscillatory behavior, while in the zigzag direction we appear to have an oscillatory behavior for small values of L and a linear behavior for large values of L. However, note that a certain variation in the tensile strength is expected in a dynamical simulation, especially when the temperature it not zero. Hence, it is likely that this is just random variation. In (b) we have the tensile strength for the structures shown in Fig. 2(b), plotted against the graphene/BN concentration. For the case where we fix the sheet size and vary the graphene/BN concentration, it is not possible to identify a well-defined pattern of behavior in both zigzag and armchair directions. Therefore, these results appear to corroborate our previous assertion that the change in values between simulations is just random variation

* leonardo@fisica.ufrn.br

[1] O. F. dos Santos and S. Azevedo, Physica E: Low-dimensional Systems and Nanostructures **116**, 113771 (2020).

[2] M.-Q. Le, Journal of Mechanical Science and Technology **28**, 4173 (2014).

[3] A. P. Thompson, S. J. Plimpton, and W. Mattson, The Journal of chemical physics **131**, 154107 (2009).
